# Biochemical characterisation and production kinetics of high molecular-weight (HMW) putative antibacterial proteins of insect pathogenic *Brevibacillus laterosporus* isolates

**DOI:** 10.1186/s12866-024-03340-2

**Published:** 2024-07-13

**Authors:** Tauseef K. Babar, Travis R. Glare, John G. Hampton, Mark R. H. Hurst, Josefina Narciso

**Affiliations:** 1 Bioprotection Research Centre, Lincoln University, Lincoln, Canterbury, 7647 New Zealand; 2 Department of Entomology, Faculty of Agricultural Sciences and Technology, Bahauddin Zakariya University, Multan, 60000 Pakistan; 3 Faculty of Agriculture and Life Sciences, Lincoln University, Lincoln, Canterbury, 7647 New Zealand; 4 Resilient agriculture, AgResearch, Lincoln Research Centre, Christchurch, New Zealand

**Keywords:** Antibacterial proteins, Biochemical characterisation, *Brevibacillus laterosporus*, High molecular-weight, Insect pathogenic isolates, Production kinetics, Spontaneous induction

## Abstract

**Background:**

Bacterial genomes often encode structures similar to phage capsids (encapsulins) and phage tails which can be induced spontaneously or using genotoxic compounds such as mitomycin C. These high molecular-weight (HMW) putative antibacterial proteins (ABPs) are used against the competitive strains under natural environment. Previously, it was unknown whether these HMW putative ABPs originating from the insect pathogenic Gram-positive, spore-forming bacterium *Brevibacillus laterosporus* (*Bl*) isolates (1821L, 1951) are spontaneously induced during the growth and pose a detrimental effect on their own survival. Furthermore, no prior work has been undertaken to determine their biochemical characteristics.

**Results:**

Using a soft agar overlay method with polyethylene glycol precipitation, a narrow spectrum of bioactivity was found from the precipitated lysate of *Bl* 1951. Electron micrographs of mitomycin C- induced filtrates showed structures similar to phage capsids and contractile tails. Bioactivity assays of cell free supernatants (CFS) extracted during the growth of *Bl* 1821L and *Bl* 1951 suggested spontaneous induction of these HMW putative ABPs with an autocidal activity. Sodium dodecyl sulphate-polyacrylamide gel electrophoresis of spontaneously induced putative ABPs showed appearance of ~ 30 kDa and ~ 48 kDa bands of varying intensity across all the time intervals during the bacterial growth except in the initial hours. Statistically, spontaneously induced HMW putative ABPs of *Bl* 1951 exhibited a significant decrease in the number of viable cells of its producer strain after 18 h of growth in liquid. In addition, a significant change in pH and prominent bioactivity of the CFS of this particular time period was noted. Biochemically, the filtered supernatant derived from either *Bl* 1821L or *Bl* 1951 maintained bioactivity over a wide range of pH and temperature.

**Conclusion:**

This study reports the spontaneous induction of HMW putative ABPs (bacteriocins) of *Bl* 1821L and *Bl* 1951 isolates during the course of growth with potential autocidal activity which is critically important during production as a potential biopesticide. A narrow spectrum of putative antibacterial activity of *Bl* 1951 precipitate was found. The stability of HMW putative ABPs of *Bl* 1821L and *Bl* 1951 over a wide range of pH and temperature can be useful in expanding the potential of this useful bacterium beyond the insecticidal value.

**Supplementary Information:**

The online version contains supplementary material available at 10.1186/s12866-024-03340-2.

## Introduction

Bacteriophages or phages are viruses that exclusively infect bacteria [1]. After infecting its host bacterium, a phage can follow two alternative cycles of replication, lytic or lysogenic [2, 3]. Lysogeny, a state in which the phage genome either remains in the host as a plasmid or integrating into the host chromosome in a form known as “prophage” is widespread among bacterial populations [4, 5]. The bacterium incorporating a prophage is called a “lysogen” [6]. Often, selective pressure can degrade the prophages to genetically defective forms [7, 8] and trap them in the host chromosome through recombination and/or deletion, and gradually decay [9], becoming inactive in terms of cell lysis, phage particle production, and plaque formation [10, 11]. Typically, these prophage fragments are referred to as cryptic or defective prophages or tailocins [12, 13]. Tailocins are phage derived antibacterial structures (bacteriocins) and similar to phages are antagonistic to the closely related producer strains [14]. Their killing mechanism involves binding with specific receptors [15, 16]. Based on their molecular weight (low molecular-weight (LMW) or high molecular-weight (HMW)), biochemical features, host range, and killing mechanism, bacteriocins represent a broad class of antagonistic substances that vary considerably [17, 18]. The HMW bacteriocins include encapsulins [19] and F-type tailocin (flexible) [20] or R-type tailocin (contractile phage tail-like structure) [21]. Encapsulins, a novel class of antibacterials (bacteriocins), structurally resembling to phage capsids has been reported from different bacteria [19, 22–26]. Structurally, bacteriocins can be linear or globular and the arrangement of the amino acids sequence and formation determine their bactericidal activity, sensitivity towards enzymes, solubility, and stability at different pHs and temperatures [27, 28]. Therefore, bacteriocins can be subjected to a range of biochemical characterisation assays.

Lysogens are usually very stable and the cell remains in this state after multiple divisions, but sometimes they can revert back to the lytic pathway due to prophage induction [29, 30]. Tailocins like phages are inducible under the influence of various environmental factors, such as DNA damage by ultraviolet (UV) radiation or use of genotoxic compounds (mitomycin C) that induces the host’s SOS response [31, 32], or it can also occur spontaneously in the absence of an external trigger in a process called “spontaneous prophage induction” (SPI) [33].

Microbial pesticides based on Gram-positive bacteria especially *Bacillus thuringiensis* (*Bt*) and *Lysinibacillus sphaericus* are the most widely used biopesticides around the world [34, 35] but several insect pests have evolved resistance to these pesticides or transgenic crops that express *Bt* toxins [36–38]. There are reports that at least 27 pest species have developed resistance to the commercially used *Bt* based products [39, 40]. Biopesticides synthesised from *L. sphaericus* have been extensively used against the dipterous insect pests especially mosquitoes [41]. Since 1994, both laboratory and field populations of *Culex pipiens* complex mosquitoes have evolved resistance to these microbial pesticides [42, 43]. This tremendous rise of resistance in insect pests against microbial pesticides necessitated the search for novel isolates or strains that can serve as a panacea to the humanity. Fortunately, another Gram-positive, spore forming, and often entomopathogenic bacterium, *Brevibacillus laterosporus* (*Bl*), of *Brevibacillus brevis* phylogenetic cluster holds a promising potential in insect pest management [44, 45]. Different insect pathogenic isolates of this useful bacterium are being considered for biopesticides development [46] due to their non-lethal effects to natural enemies i.e., predators and parasites [47], potential to produce numerous antibacterial and antifungal compounds [48], and insecticidal proteins [49]. Furthermore, on account of its wide spectrum activity against insect pests [50, 51], there has been an exponential increase in the registration of *Bl* based pesticidal proteins as patents [52–55]. In New Zealand, three insect pathogenic isolates *Bl* 1821L, *Bl* 1951, and *Bl* Rsp exhibiting larvicidal activity against the diamondback moth and mosquitoes have been isolated and characterised [56–58]. Currently, two isolates *Bl* 1821L and *Bl* 1951 are under development as a biopesticide but unfortunately often experience a stunted growth in culture which was hypothesised to be due to the activity of *Tectiviridae* phages [49, 59]. Subsequent experimental work could not substantiate this hypothesis [60] and the putative antibacterial and autocidal activity of the mitomycin C- induced cultures of *Bl* 1821L and *Bl* 1951 isolates was found to be associated with a phage tail-like bacteriocin (~ 48 kDa) [61] and Linocin M18 protein (31.4 kDa) [26]. *Brevibacillus* species are a large source of antimicrobial peptides (AMPs) and >30 AMPs with varied antimicrobial activity have been isolated from different species [62]. However, only example of the LMW bacteriocins with potential self-killing activity originating from the insect pathogenic *Bl* isolates can be found in literature [63]. In the past no research work has been undertaken to assess the inherent potential of the spontaneously induced HMW putative antibacterial proteins (ABPs) of *Bl* 1821L and *Bl* 1951 isolates during the course of growth and their effect on the host survival. Furthermore, prior to it the HMW putative ABPs belonging to the insect pathogenic bacteria has not been biochemically characterised.

In this study, for the first time we identified the phenomenon of spontaneous induction of HMW putative ABPs of *Bl* 1821L and *Bl* 1951 isolates while determining the production kinetics of these putative ABPs under normal cultivating conditions at various time intervals and their effect on the growth of bacteriocinogenic isolates was also examined. Furthermore, we also characterised the biochemical features of the crude lysate harbouring the HMW putative ABPs of the insect pathogenic *Bl* 1821L and *Bl* 1951 isolates.

## Methods

### Bacterial isolates and culture conditions

Entomopathogenic isolates *Bl* 1821L and *Bl* 1951 used in this study were originally discovered from the *Brassica* seeds [56] and maintained at -80 °C using the cryobeads bacterial preservation system (Technical Service Consultants Limited, UK) in the Microbial Culture Collection of Bioprotection Research Centre (BPRC), Lincoln University, New Zealand. For experimentation, the isolates were cultured in Luria-Bertani (LB Miller, Sigma, St. Louis, MI, USA) broth overnight on an orbital shaker (Conco, TU 4540, Taibei, Taiwan) at 250 rpm and 30 °C.

### Mitomycin C induction of putative ABPs

Putative ABPs residing in the chromosomes of *Bl* 1821L (NZ_CP033464.1) and *Bl* 1951 (RHPK01000003, contig 1) [49] were induced using the mitomycin C as outlined by [60, 61]. Five hundred microlitre of overnight culture was used to inoculate 25 mL of LB broth and left to shake on an orbital shaker at 250 rpm and 30 °C for 10–12 h (until culture attained turbidity). At this point, mitomycin C (Sigma, Sydney, NSW, Australia) at a concentration of 1 µg/mL [61] and 3 µg/mL [60] was added into *Bl* 1821L and *Bl* 1951 cultures respectively and placed overnight on an orbital platform for incubation at 40 rpm and room temperature (24 °C). Finally, optical density (OD) of the culture was recorded at 600 nm through an Ultrospec-10 spectrophotometer (Amersham Biosciences, Amersham, UK). The appearance of clean culture or accumulation of bacterial debris and a drastic decrease in OD_600nm_ indicated the lysis of cells (induction of putative ABPs). After induction, the culture was centrifuged at 16,000 x g for 10 min and the supernatant was filtered through a 0.22 μm filter (Merck Millipore, UK). The cultures without addition of mitomycin C served as a control.

### Disc diffusion assay test of mitomycin C- induced and uninduced filtrates

Mitomycin C- induced and uninduced (without mitomycin C) filtrates of *Bl* 1821L and *Bl* 1951 were tested for their potential antibacterial activity against *Bl* 1821L and *Bl* 1951 as the indicator isolates using a modified protocol [64] of [65]. In short, overnight culture of the indicator isolate was swabbed over the surface of an LB agar plate and left to dry for 10–15 min at 24 °C. Next, 80 µL of filtrate with or without mitomycin C induction was pipetted onto a sterile 8 mm diameter paper disc (ADVANTEC, Niigata, Japan). For the negative control, 10 x Tris buffer saline (TBS, 25 mM Tris-HCl, 130 mM NaCl, pH 7.5) was used. Antibacterial activity of the filtered supernatants of *Bl* 1821L and *Bl* 1951 isolates was measured through the diameter (mm) of the zone of inhibition (including the diameter of the disc). Disc diffusion assays were performed in triplicate.

### Transmission electron microscopy (TEM) of crude lysates

For TEM, protocol of [66] with some modifications as previously outlined by [26] was followed. Seven milliliter of mitomycin C- induced filtrate was ultracentrifuged at 151,263 x g for 70 min and 4 °C. The supernatant was decanted and the pellet resuspended gently in 150 µL of 25 mM TBS. After negative staining with 0.7% uranyl acetate (UA, pH 5.0), 5 µL of concentrate was applied to a freshly glow-discharged plastic-coated hydrophilic 200 mesh EM grid (ProSciTech; Thuringowa, Australia). Next, the sample was examined by TEM using a Morgagni 268D (FEI, Hillsboro, OR, USA) electron microscope at a magnification of 18,000–25,000 with an operating voltage of 80 KeV and the micrographs were taken with the aid of a TENGRA camera.

### Soft-agar overlay method with polyethylene glycol (PEG) precipitation

Soft agar overlay method of [11] with PEG precipitation of mitomycin C- induced filtrates and performing serial dilution assay is being used to differentiate between the antagonistic activity of putative bacteriophages and bacteriocins. Based on the potent bioactivity of mitomycin C- induced filtrates in disc diffusion assay and TEM examination, we used this method with some modifications as outlined earlier by [61] to identify the nature of putative ABPs present in the CFS of *Bl* 1951. Briefly, after mitomycin C induction, 1 M NaCl and PEG 8000 (10%) were mixed with the filtered supernatant and the mixture was repeatedly inverted until all the ingredients had been dissolved. This sample was then incubated in an ice bath for 60 min and centrifuged at 16,000 x g for 30 min. The resultant supernatant was removed carefully and the pellet was resuspended in 1/10th volume of the original supernatant volume of buffer (10 mM Tris, 10 mM MgSO_4_, pH 7.0). To sterilize the supernatant from PEG residues, an equal volume of chloroform was added and the mixture was vortexed for 10–15 s followed by centrifugation at 16,000 x g for 10 min. The resultant upper aqueous phase of mixture was transferred to a fresh microfuge tube. This extraction process was repeated until no white interface between the aqueous and organic phases was visible. Next, soft agar (0.5%) overlay was prepared and kept in a water bath at 55–60 °C before use in the bioactivity assay of the precipitated filtrate of *Bl* 1951 in serial dilutions following the protocol of [61]. For the negative control, 10 x TBS was spotted on the LB agar plates. Antagonistic potency of PEG 8000 precipitated filtrate of *Bl* 1951 isolate was determined by measuring the diameter (mm) of the clearing zone (lysis) at the inoculation point. Spot agar overlay assay was performed in triplicate.

### Antibacterial spectrum of PEG 8000 precipitated filtrate

Antibacterial spectrum of PEG 8000 precipitated filtrate of *Bl* 1951 after mitomycin C induction against itself and various Gram-positive bacteria (*Bl* 1821L, *Bl* Rsp, *Bl* CCEB 342, *Bl* NRS 590, *Bacillus megaterium* 3 − 2, *B. megaterium* S1, *B. subtilis* Tp5, *Carnobacterium maltaromaticum* 3-1, *Fictibacillus rigui* FJAT 46895, *Oceanobacillus* sp. R-31213, *Oerskovia enterophila* 3-3, *Paenibacillus* sp. 15.12.1) was defined according to method of [11] as outlined earlier by [61]. All these strains are preserved at -80 °C in LB broth supplemented with 50% glycerol in the Microbial Culture Collection of BPRC, Lincoln University, New Zealand. For the negative control, 10 x TBS was spotted on the LB agar plates. Antibacterial activity of PEG 8000 precipitated filtrate was determined by measuring the diameter (mm) of the clearing zone (lysis) at the inoculation point. Spot agar overlay assay was performed in triplicate.

### Effect of enzymes on the bioactivity of crude putative ABPs

Mitomycin C- induced culture of *Bl* 1821L or *Bl* 1951 was centrifuged at 16,000 x g for 10 min at 4 °C and the supernatant was filtered through a 0.22 μm filter. CFS was treated with three enzymes; catalase, protease, and proteinase K (Sigma-Aldrich, St. Louis, MO, USA) at a final concentration of 1 mg/mL to evaluate their effect on the bioactivity of crude putative ABPs. All the enzymes were prepared by dissolving in 10 mM sodium phosphate buffer (pH 7.5). The preparations were then incubated at 30 °C for 6 h followed by heating at 85 °C for 5 min to terminate the enzymatic activity. For the positive control, CFS and 10 x TBS without enzymes was used.

Antibacterial activity of *Bl* 1821L and *Bl* 1951 crude preparations containing the putative phage head and tail-like structures was determined through the Kirby-Bauer disc diffusion assay with some modifications as outlined above [64, 65]. Antagonistic action of crude lysate of *Bl* 1821L and *Bl* 1951 treated with and without enzymes was examined by measuring the diameter (mm) of the zone of inhibition (including the diameter of the paper disc). Disc diffusion assay was performed in triplicate.

### Effect of temperature on the bioactivity of crude putative ABPs

Thermal stability of crude preparations of *Bl* 1821L and *Bl* 1951 harbouring the putative phage head and tail-like structures was evaluated by treating the filtered supernatant at 70 °C, 80 °C, 90 °C, and 100 °C for 60 min. The activity of crude putative ABPs of *Bl* 1821L and *Bl* 1951 was also evaluated after autoclaving at 121 °C for 15 min. The samples were immediately chilled on an ice after heating and assayed for bioactivity through the Kirby-Bauer disc diffusion test with some modifications as mentioned earlier [64, 65] against vice versa isolate as the host bacterium. Aliquots (1 mL) of CFS were also exposed to 4 °C and − 20 °C for 30 days to determine their stability in storage. For the positive control, mitomycin C- induced *Bl* 1821L and *Bl* 1951 filtered supernatant without heating was used. Each treatment was replicated thrice and the average value of the diameter of zone of inhibition (mm) was recorded. Three independent set of experiments were performed. Mean values of all experiments were subjected to analysis of variance (ANOVA) using the software Statistix 8.1 and to determine the significance differences among the treatments, Tukey’s honest significant difference (HSD) all pairwise comparison test at α = 0.05 level was used [67]. Results were considered significant if P-value was less than 0.05 (*P* < 0.05).

### Effect of pH on the bioactivity of crude putative ABPs

To determine the effect of pH on the bioactivity of crude preparations of *Bl* 1821L and *Bl* 1951 containing the putative phage head and tail-like structures, the pH of CFS was adjusted to 2.0, 4.0, 6.0, 8.0, 10.0, and 12.0 using either hydrochloric acid (Merck, New Zealand) or sodium hydroxide (Merck, New Zealand). The residual activity of all pH treated samples was evaluated using the Kirby-Bauer disc diffusion assay with some modifications as described above [64, 65] against vice versa isolate as the host bacterium. Mitomycin C- induced *Bl* 1821L and *Bl* 1951 filtrates without any alteration of pH served as a positive control. Each treatment was replicated thrice and the average value of the diameter of zone of inhibition (mm) was recorded. Three independent set of experiments were performed. Mean values of all experiments were subjected to ANOVA using the software Statistix 8.1 and to determine significance differences among the treatments, Tukey HSD all pairwise comparison test at α = 0.05 level was used [67]. Results were considered significant if P-value was less than 0.05 (*P* < 0.05).

### Production kinetics of putative ABPs

Cultures of insect pathogenic isolates *Bl* 1821L and *Bl* 1951 preserved at -80 °C in LB broth supplemented with 50% glycerol were used for streaking on LB agar plates to cultivate a primary culture. An LB agar plate was further streaked from a colony of *Bl* 1821L and *Bl* 1951 primary culture to obtain the secondary culture. A single colony of each secondary culture was picked to inoculate 5 mL of sterile LB broth in a universal vial. The *Bl* 1821L and *Bl* 1951 inoculated vials were placed on an orbital shaker overnight at 30 °C and 250 rpm. One millilitre of an overnight culture of the host bacterium *Bl* 1821L and *Bl* 1951 was transferred into 25 mL LB broth and placed on an orbital shaker to propagate at 250 rpm and 30 °C. Two separate flasks were used for each treatment which were taken out for assessment at the following time intervals 3, 6, 12, 18, 24, 36, 48, 60, 72, 96, 120, 144, 168, 192, 216, and 240 h. At each time point, a sample of 1 mL was aseptically drawn from each treatment to prepare tenfold serial dilutions (10^− 1^ to 10^− 6^). One hundred microlitre of each dilution was transferred onto an LB agar plate and the spread culture was left to dry for 10–15 min at 24 °C. Two replicate LB agar plates were inoculated with the drawn sample and kept in an incubator at 30 °C. The *Bl* 1821L and *Bl* 1951 colonies were counted with the help of a colony counter (Cole-Palmer™ Stuart™, Thermo Fisher Scientific, UK) after 48–72 h of spreading and converted into CFU/mL.

The *Bl* 1821L and *Bl* 1951 cultures were then independently centrifuged at 16,000 x g for 10 min at 4 °C and the supernatants were filtered through a 0.22 μm filter. The pH of *Bl* 1821L and *Bl* 1951 CFS extracted at each interval was recorded with a pH meter (Orion star A211, Thermofisher, Waltham, MA, USA).

Antibacterial activity of each time interval filtered supernatant of *Bl* 1821L and *Bl* 1951 was tested against the indicator isolates *Bl* 1821L and *Bl* 1951 through the Kirby-Bauer disc diffusion assay with some modifications as outlined above [64, 65]. The *Bl* 1821L and *Bl* 1951 produced putative ABPs antagonistic activity against indicator isolates was examined by measuring the diameter (mm) of the zone of inhibition (including the diameter of the paper disc). The production kinetics experiment was undertaken in triplicate, from where the data of assessed parameters, CFU/mL, pH of CFS, and diameter (mm) of clearing zones (lysis) from each of the treatments were pooled. Three independent set of experiments were performed. CFU/mL of each experiment was converted into log_10_ CFU/mL and the pooled data was subjected to ANOVA using the Genstat 20th edition. Value of least significant difference (LSD) at 5% of the means of each assessed parameter was used to determine the significance or insignificance of the data.

### Sodium dodecyl sulphate-polyacrylamide gel electrophoresis (SDS-PAGE) of spontaneously induced putative ABPs

CFS of *Bl* 1821L and *Bl* 1951 spontaneously induced HMW putative ABPs at various time intervals were ultracentrifuged at 151,263 x g for 70 min at 4 °C. High speed centrifugation concentrated the spontaneously induced HMW putative ABPs of *Bl* 1821L and *Bl* 1951 which were run on SDS-PAGE for visualisation according to the protocol of [68]. As a standard, 10 µL of protein ladder (BIO-RAD, Precision Plus Protein™ Standards, Hercules, CA, USA) was loaded. For electrophoresis, the gel was run for 50 min at 200 volts and before staining it was washed four times with dH_2_O. RAMA staining method of [69] was used to stain the gel. The electrophoretic pattern of spontaneously induced HMW putative ABPs was compared with the mitomycin C- induced filtrates of *Bl* 1821L and *Bl* 1951 serving as a control.

## Results

### Disc diffusion assay of mitomycin C- induced and uninduced filtrates

A prominent decrease in OD_600 nm_ of the grown cultures of *Bl* 1821L (1.26 to 0.20) and *Bl* 1951 (1.41 to 0.71) was noted 24 h after treatment with mitomycin C which was an indication of cell lysis due to induction. Assessment of the CFS of mitomycin C- induced and uniduced cultures of *Bl* 1821L and *Bl* 1951 showed the presence of putative ABPs by producing the prominent clearing zones (lysis) not only on the lawns of producer but also vice versa isolate (Supplementary (S) Information Figure S1). However, the inhibition zones produced by the uninduced filtrates were slightly smaller than the others (Figure S1).

### TEM examination of crude filtrates

**Fig. 1:**
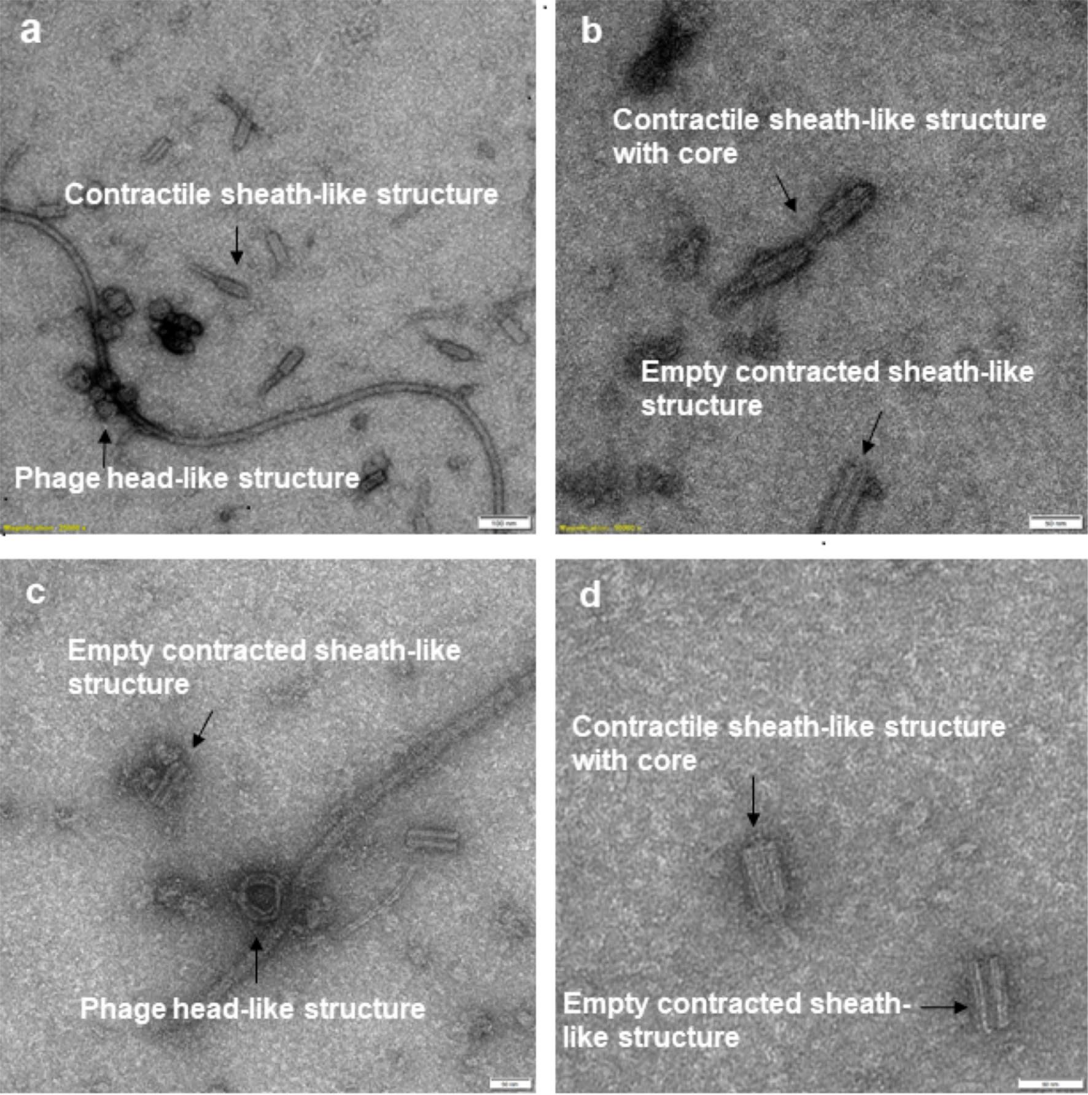
Transmission electron microscope images of the putative phage structural parts observed in the mitomycin C- induced flitrates of *Bl* 1821L (1a, 1b) and *Bl* 1951 (1c, 1d). Structures resembling to phage head (capsid) and incomplete phage particles with an empty contracted sheath, contractile tail sheath, and contractile sheath with cores were seen (Fig. 1a- 1d). Scale bar = Fig. 1a = 100 nm, Fig. 1b- 1d = 50 nm

Electron micrographs of mitomycin C- induced filtrates from *Bl* 1821L (Fig. 1a, 1b) or *Bl* 1951 (Fig. 1c, 1d) showed the presence of alike structures. Putative phage structural parts resmbling the phage head (capsid) and incomplete phage particles with an empty contracted sheath, contractile tail sheath, and contractile sheath with cores were seen (Fig. 1a- 1d).

### Antagonistic activity of PEG 8000 precipitated filtrate in the soft agar ovelay method

Bioactivity assay of the PEG 8000 precipitated filtrate of *Bl* 1951 in serial dilutions exhibited no antagonism against the producer isolate at most levels of dilution but a prominent clearing zone (lysis) was noted from full strength (FS) to 10^− 1^ level of dilution when *Bl* 1821L was used as the indicator isolate (Table 1, Figure S2a, S2b). Additionally, the clearing zone (lysis) were picked to inoculate a propagating culture of *Bl* 1951 and then spotted onto a fresh soft agar overlay seeded with the same indicator isolate but no effect was found which suggested the existence of bacteriocins in the PEG 8000 precipitated filtrate of *Bl* 1951 rather than bacteriophages. In the control treatment, where 10 x TBS buffer was spotted on the lawns of indicator isolates *Bl* 1951 and *Bl* 1821L, no bioactivity was observed (Figure S2c, S2d).

**Table 1:** Antagonistic activity of PEG 8000 precipitated filtrate of *Bl* 1951 against *Bl* 1951 and *Bl* 1821L

*Bl* 1951 as the host bacterium		*Bl *1821L as the host bacterium	
**Dilution level**	**Zone of inhibition** **diameter (mm)**	**Dilution level**	**Zone of inhibition** **diameter (mm)**
*FS	-**	FS	12.5
10^− 1^	-	10^− 1^	13.5
10^− 2^	-	10^− 2^	-
10^− 3^	-	10^− 3^	-
10^− 4^	-	10^− 4^	-
10^− 5^	-	10^− 5^	-
10^− 6^	-	10^− 6^	-
10^− 7^	-	10^− 7^	-
10^− 8^	-	10^− 8^	-
Control	-	Control	-

* FS = Full strength

** - = No zone of inhibition

While determining the antibacterial spectrum of the PEG 8000 precipitated filtrate of *Bl* 1951 after mitomycin C induction, varying levels of bioactivity were noted against all the *Bl* strains used in this study except for the producer strain (itself) and no lethality was noted against the other evaluated Gram-positive bacteria (Table 2, Figure S3).

**Table 2:** Antibacterial activity of PEG 8000 precipitated filtrate of *Bl* 1951 against itself and various Gram- positive bacteria

Host bacterium	Host bacterium isolate/strain	Sensitivity to PEG 8000 precipitated filtrate of *Bl *1951
*Bacillus megaterium*	3- 2	-
*Bacillus megaterium*	S1	-
*Bacillus subtilis*	EM-13 (Tp5)	-
*Brevibacillus laterosporus*	1951	-
*Brevibacillus laterosporus*	1821L	+++
*Brevibacillus laterosporus*	Rsp	+++
*Brevibacillus laterosporus*	NRS 590	+++
*Brevibacillus laterosporus*	CCEB 342	++
*Brevibacillus laterosporus*	NCIMB	+
*Carnobacterium maltaromaticum*	3- 1	-
*Fictibacillus rigui*	EM-14 (FJAT 46895)	-
*Oceanobacillus* spp.	EM-12 (R-31213)	-
*Oerskovia enterophila*	3-3	-
*Paenibacillus* spp.	15.12.1	-

Note: Antibacterial effect of PEG 8000 precipitated filtrate of *Bl* 1951 against itself and various Gram- positive bacteria was graded on the basis of the diameter of the zone of inhibition (mm) on the lawn of the indicator strain

- = No potential antibacterial activity

+ = Low level of antibacterial activity

++ = Medium level of antibacterial activity

+++= High level of antibacterial activity

### Effect of enzymes on the bioactivity of crude putative ABPs

The *Bl* 1821L and *Bl* 1951 filtered supernatants that contained the putative phage head and tail-like structures after treatment with the proteolytic enzymes (proteinase K and protease) lost their antagonistic activity, validating their proteinaceous nature (Table S1). Catalase treatment of the mitomycin C- induced filtrates of *Bl* 1821L and *Bl* 1951 did not affect the antibacterial activity, demonstrating that the developed clearing zones (lysis) on the lawns of indicator isolates were not due to action of hydrogen peroxide (H_2_O_2_). The crude preparations of *Bl* 1821L and *Bl* 1951 harbouring the putative ABPs in the control treatment (without enzymes) maintained the antagonistic activity (Table S1).

### Effect of pH on the bioactivity of crude putative ABPs

Antibacterial activity of the crude *Bl* 1821L preparations containing the putative ABPs against the indicator isolate *Bl* 1951 persisted at all the evaluated pHs except for pH 12.0. No zone of inhibition was observed at pH 12.0 and it differed significantly from the bioactivity at other pH values (Table 3, Figure S4). However, with the exception of pH 2.0, the CFS of *Bl* 1951 containing the putative ABPs demonstrated more pronounced effect against the indicator isolate *Bl* 1821L at all the adjusted pH values. Statistically, putative ABPs of *Bl* 1951 exhibited more potent activity at pH 10.0 followed by pH 8.0, pH 6.0, pH 4.0, and pH 12.0 (Table 3, Figure S4).

**Table 3:** Effect of pH on the bioactivity of filtered supernatant of *Bl* 1821L and *Bl* 1951 harbouring the putative antibacterial proteins (bacteriocins) against the indicator isolates *Bl* 1951 and *Bl* 1821L

pH	Zone of inhibition diameter (mm)	
	*Bl* 1951 as the host bacterium	*Bl* 1821L as the host bacterium
2	11.3 ± 0.58 b	00.0 ± 0.00 d
4	11.0 ± 0.00 b	11.67 ± 0.76 c
6	11.7 ± 0.58 b	20.7 ± 0.29 b
8	11.7 ± 0.58 b	21.0 ± 0.50 b
10	11.7 ± 0.29 b	21.7 ± 0.76 ab
12	00.0 ± 0.00 c	10.7 ± 0.58 c
Control	15.0 ± 0.50 a	23.0 ± 0.50 a
Critical value for comparison (α = 0.05)	1.1858	1.6770

Note:  Effect of different pHs on the bioactivity of putative antibacterial proteins was compared using the Tukey HSD all pairwise-comparison test at α = 0.05. Mean values of the diameter of zone of inhibition (mm) of three experiments are presented with the standard of error (Means ± SE). Values with the different letters are significantly different as determined by Tukey’s HSD all pairwise-comparison (*P* < 0.05)

### Effect of temperature on the bioactivity of crude putative ABPs

The filtered supernatant of *Bl* 1821L harbouring the putative ABPs extracted after mitomycin C induction retained thermal stability at 70 °C, 80 °C, and 90 °C except at 100 °C and 121 °C where no clearing zones (lysis) were observed. Statistically, bioactivity of the putative ABPs at 70 °C and 80 °C was similar to the control treatment but differed significantly from the treatment at 90 °C (Table 4, Figure S5). The *Bl* 1821L putative ABPs kept at 4 °C and − 20 °C for 30 days sustained their antagonism against the indicator isolate *Bl* 1951. However, exposure to -20 °C reduced the bioactivity of filtered supernatant of *Bl* 1821L (data not shown).

**Table 4:** Effect of temperature on the bioactivity of filtered supernatant of *Bl* 1821L and *Bl* 1951 harbouring the putative antibacterial proteins (bacteriocins) against the indicator isolates *Bl* 1951 and *Bl* 1821L

Temperature (°C)	Zone of inhibition diameter (mm)	
	*Bl* 1951 as the host bacterium	*Bl* 1821L as the host bacterium
70	15.0 ± 1.00 a	17.0 ± 1.00 b
80	14.0 ± 0.87 a	18.7 ± 0.76 ab
90	11.7 ± 0.29 b	14.3 ± 0.58 c
100	00.0 ± 0.00 c	14.7 ± 0.76 c
121	00.0 ± 0.00 c	00.0 ± 0.00 d
Control	15.8 ± 1.04 a	20.3 ± 0.58 a
Critical value for comparison (α = 0.05)	2.1469	2.0837

Note: Effect of different temperatures on the bioactivity of putative antibacterial proteins was compared using the Tukey HSD all pairwise-comparison test at α = 0.05. Mean values of the diameter of zone of inhibition (mm) of three experiments are presented with the standard of error (Means ± SE). Values with the different letters are significantly different as determined by Tukey’s HSD all pairwise-comparison (*P* < 0.05)

The crude putative ABPs of *Bl* 1951 sustained their bioactivity against the indicator isolate *Bl* 1821L at all the evaluated temperatures except at 121 °C, where no clearing zone (lysis) was observed after heating for 15 min. Statistically, putative ABPs of *Bl* 1951 exhibited more stability at 80 °C followed by 70 °C, 90 °C, and 100 °C (Table 4, Figure S5). The crude filtered supernatant maintained at 4 °C for 30 days did not lose stability when evaluated against the indicator isolate *Bl* 1821L. However, exposure to -20 °C resulted in a slight decrease in antagonism (data not shown).

### Production kinetics of *Bl* 1821L putative ABPs

In the present study, the results of bioactivity assays of the CFS derived from the *Bl* 1821L and *Bl* 1951 cultures without mitomycin C addition and the serial dilutions assay of the mitomycin C induced filtrates after PEG 8000 precipitation suggested the spontaneous induction of HMW putative ABPs. Therefore, production kinetics experiment was performed to find out whether these HMW putative ABPs are spontaneously induced during the growth and inflict a lethal effect on their own survival.

**Fig. 2:**
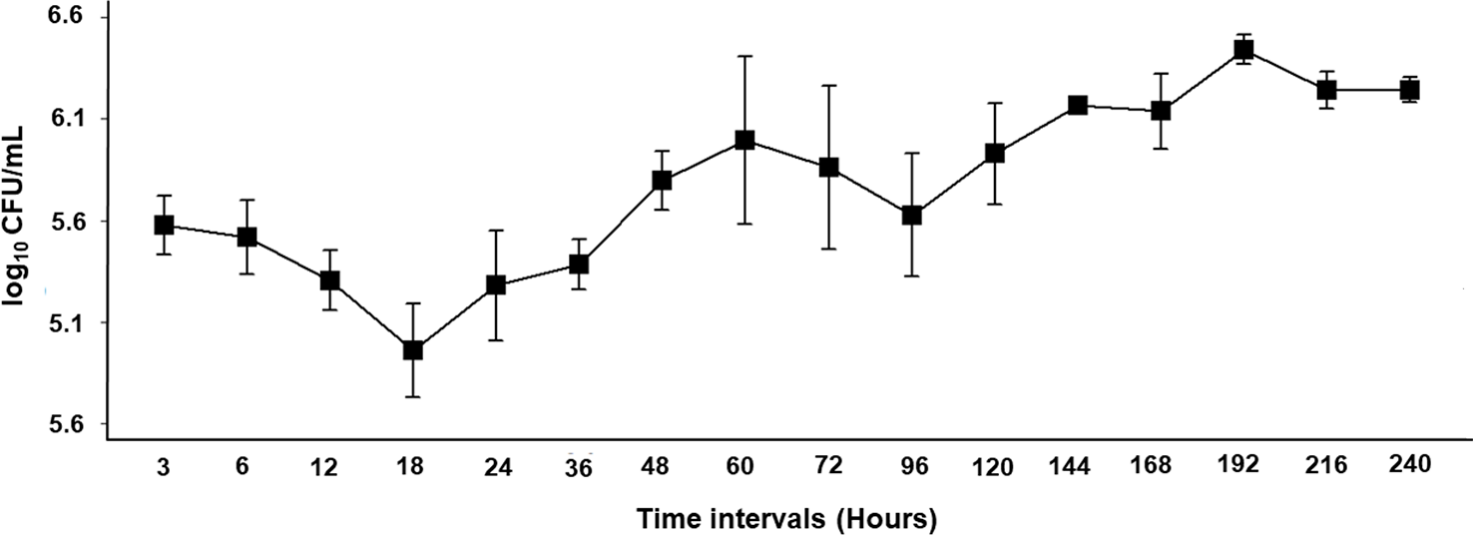
Mean values of *Bl* 1821L cells growth (log_10_ CFU/mL) at various time intervals are shown with the standard error. Mean values are averaged from the pooled data of three experiments

Antibacterial activity of filtered supernatants of *Bl* 1821L harbouring the putative ABPs against itself (autocidal) was evident 3 h after inoculation, as indicated by a small lysis zone of 11.3 mm but the highest inhibitory activity (13.7 mm) was noted with the CFS of 36 h (Table S2, Figure S6). The number of viable cells started to decline gradually and after 18 h a dip was observed (Table S2, Fig. 2). CFS extracted at this time interval (18 h) produced a lysis zone of 13.2 mm against its own lawn (Table S2, Figure S6). However, this dip in colony forming units (log_10_ CFU/mL) was not statistically significant when compared to 12–36 h of growth respectively (Table S2, Fig. 2). CFS of 36 h produced the highest clearing zone (lysis) of 13.7 mm on the lawns of producer isolate (*Bl* 1821L) which did not differ from the bioactivity of 18 h (13.2 mm) (Table S2, Figure S6). The pH of *Bl* 1821L filtered supernatants varied from 7.04 to 9.47 and it steadily increased up to 60 h but the point (18 h) where log_10_ CFU/mL demonstrated a fall indicated a significant change in pH value as compared to pH at 12 h and 36 h CFS respectively (Table S2). The *Bl* 1821L supernatants extracted at 60–72 h post inoculation exhibited the highest antagonism against *Bl* 1951, producing an inhibition zone of 15.7 mm and 15.4 mm respectively (Table S2, Figure S7). This was the point where log_10_ CFU/mL values began to decline at an insignificant level. The pH of *Bl* 1821L filtered supernatants of both the time intervals statistically did not differ from each other (Table S2). Assessments of the paper discs with 10 x TBS serving as a negative control demonstrated no antagonistic activity (Figure S6, S7).

### Production kinetics of *Bl* 1951 putative ABPs

**Fig. 3:**
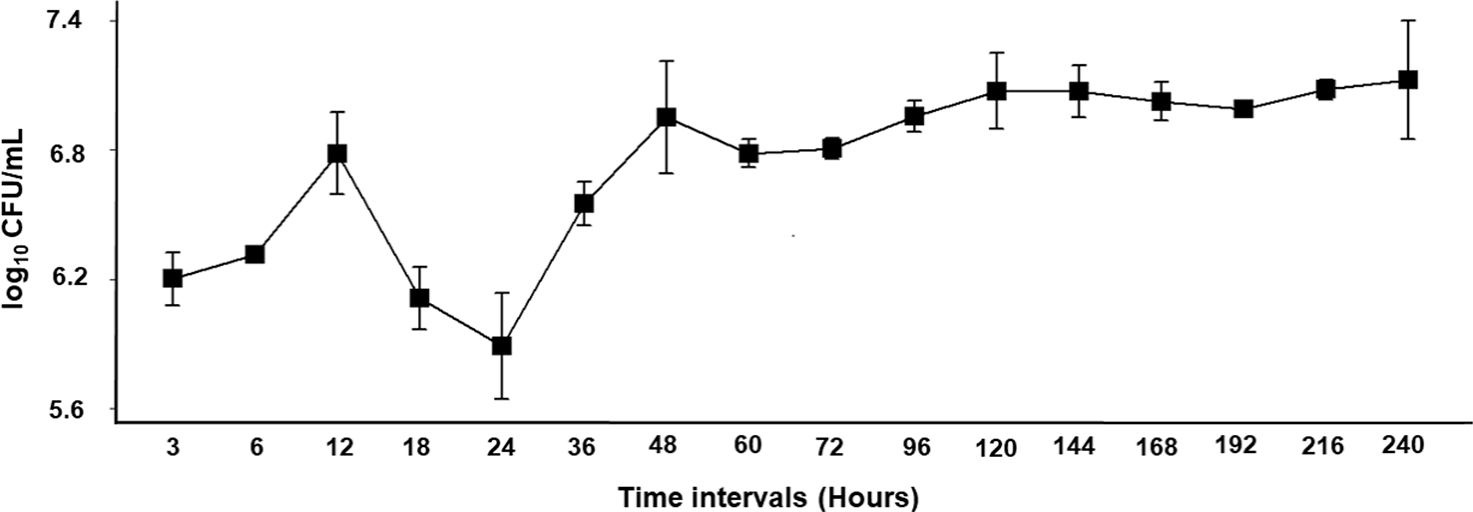
Mean values of *Bl* 1951 cells growth (log_10_ CFU/mL) at various time intervals are shown with the standard error. Mean values are averaged from the pooled data of three experiments

Antimicrobial activity of the crude CFS of *Bl* 1951 harbouring the putative ABPs against *Bl* 1951 was initiated after 12 h by producing a narrow clearing zone (lysis) of 10.7 mm but against *Bl* 1821L, the activity started to appear even from the CFS of 3 h (Table S3, Figure S8, S9). The number of viable cells abruptly fell after 18–24 h of growth (Fig. 3). The time point where the culture of *Bl* 1951 isolate experienced a dip in log_10_ CFU/mL also corresponded to the prominent antagonistic activity of CFS against *Bl* 1951 and *Bl* 1821L, where inhibitory zones of 13.3 mm and 15 mm respectively were measured (Table S3, Fig. 3, Figure S8, S9). No zone of inhibition was produced in the control treatment where 10 x TBS was used (Table S3, Figure S8, S9). All the assessed parameters at the dipping point (18 h) including log_10_ CFU/mL, pH of CFS, and the diameters of zones of inhibition against the indicator isolates *Bl* 1951 and *Bl* 1821L differed significantly from the initial periods (3–12 h) of growth (Table S3). After 48 h, a non-significant decline in the number of viable cells of *Bl* 1951 isolate was noticed (Table S3, Fig. 3). The pH of *Bl* 1951 CFS obtained at various time intervals varied from 6.95 to 9.32, increasing slowly up to 36 h and afterwards it almost remained static or slightly fluctuated (Table S3).

### SDS-PAGE analysis of spontaneously induced putative ABPs

**Fig. 4:**
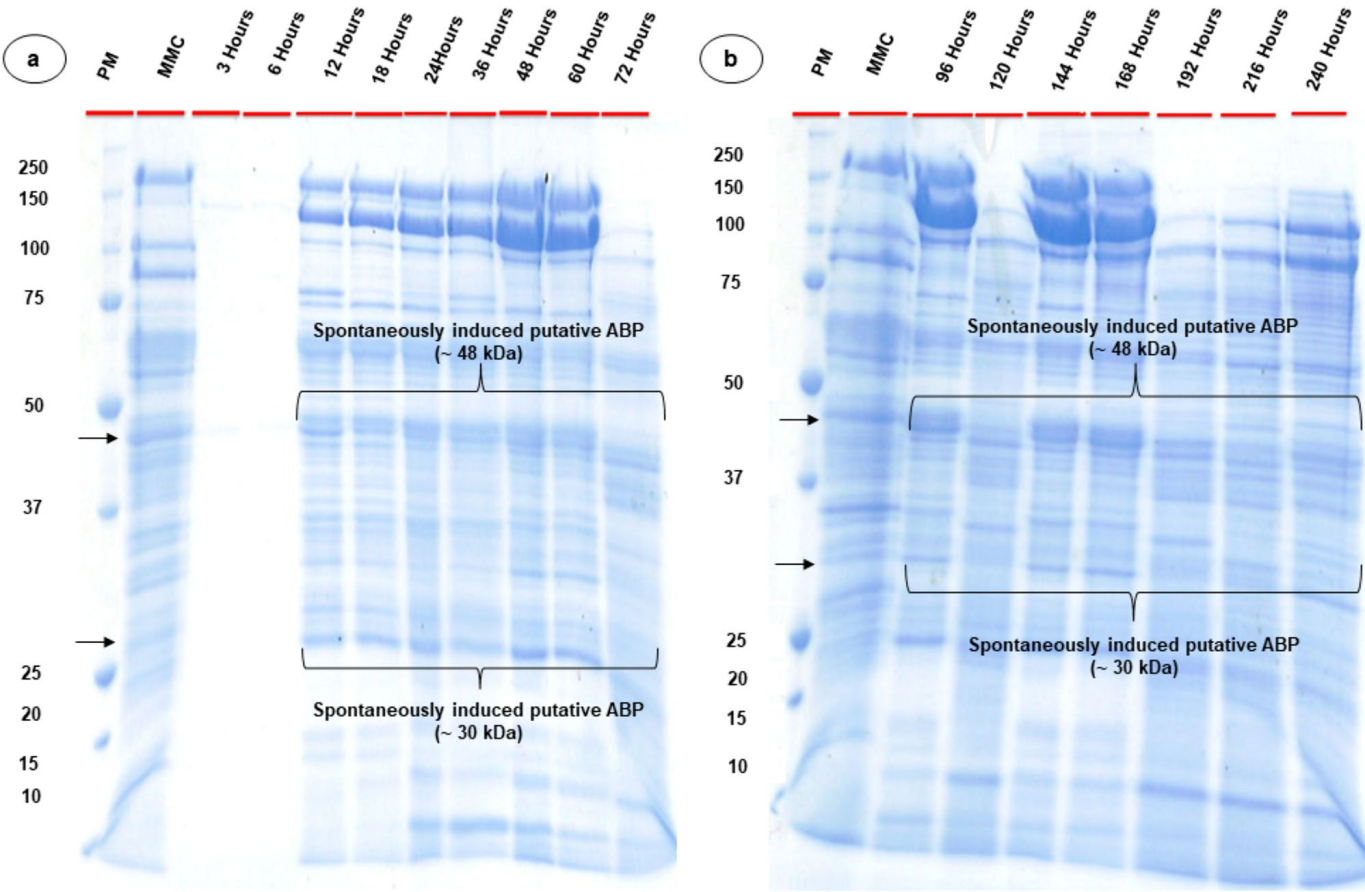
SDS-PAGE analysis of spontaneously induced putative antibacterial proteins of *Bl* 1821L across various time intervals. (**a**) shows the spontaneously induced putative antibacterial proteins after 3, 6, 12, 18, 24, 36, 48, 60, 72 h and (**b**) shows the protein bands of spontaneously induced putative antibacterial proteins after 96, 120, 144, 168, 192, 216, and 240 h of *Bl* 1821L cultivation at 30 °C and 250 rpm. Black arrows denote the mitomycin C- induced putative antibacterial proteins of ~ 30 kDa and ~ 48 kDa. PM and MMC stands for protein marker and mitomycin-C respectively

CFS of *Bl* 1821L extracted in the initial 3–6 h of growth after concentration yielded no protein bands on the SDS-PAGE (Fig. 4a), which was in agreement with the results of bioactivity assay of the CFS of the same time intervals (Table S2, Figure S6, S7). Previously, we found that two putative ABPs of 31.4 kDa and ~ 48 kDa were implicated in antibacterial activity of the isolates *Bl* 1821L and *Bl* 1951 [26, 61]. In the current study, both the identified proteins (~ 30 kDa and ~ 48 kDa) were visualised on SDS-PAGE between 12 and 240 h of *Bl* 1821L growth with varying intensity as compared to the protein bands observed in mitomycin C- induced culture (Fig. 4a, 4b).

**Fig. 5:**
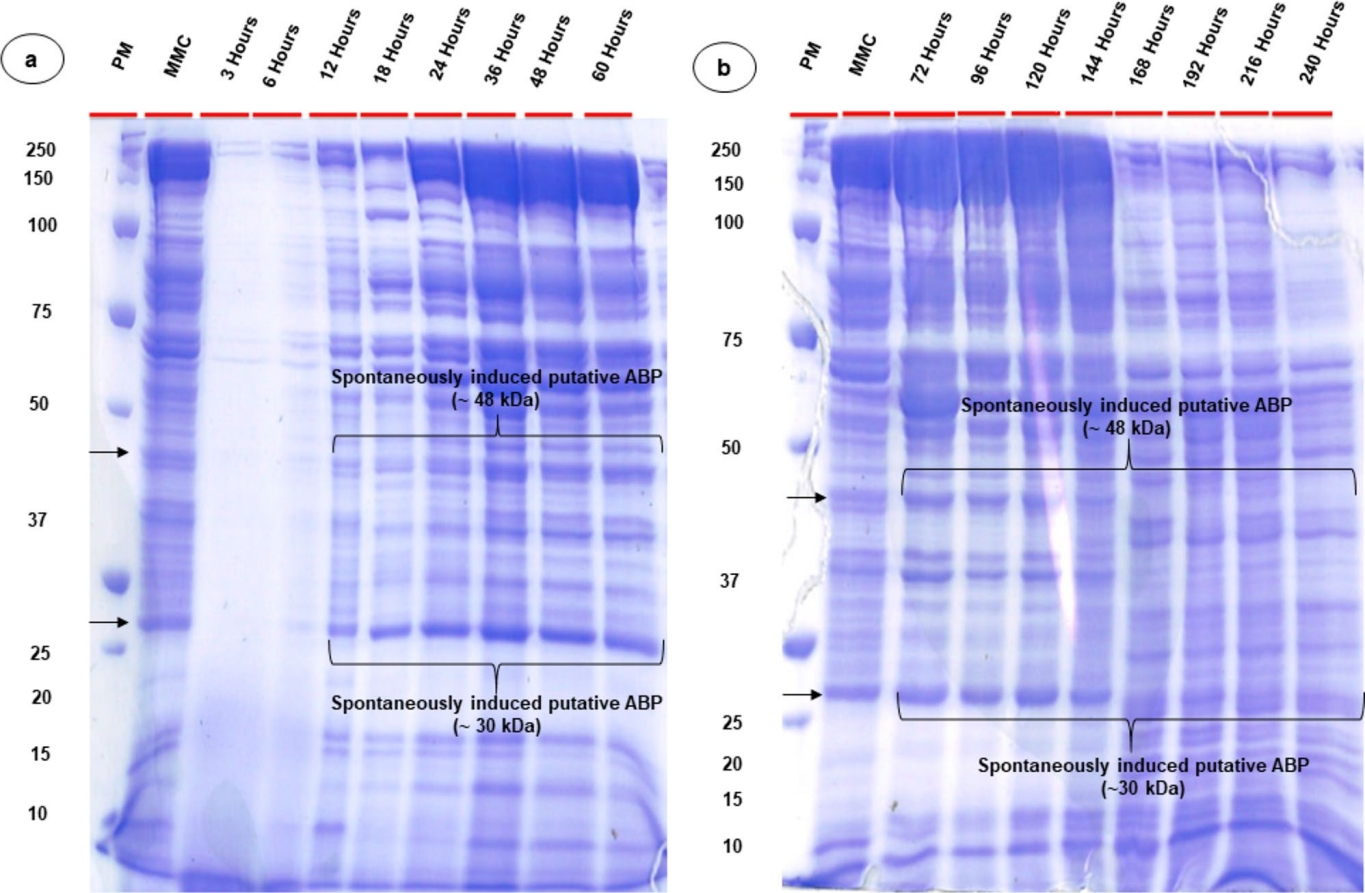
SDS-PAGE analysis of spontaneously induced putative antibacterial proteins of *Bl* 1951 across various time intervals. (**a**) shows the spontaneously induced putative antibacterial proteins after 3, 6, 12, 18, 24, 36, 48, 60, 72 h and (**b**) shows the protein bands of spontaneously induced putative antibacterial proteins after 96, 120, 144, 168, 192, 216, and 240 h of *Bl* 1951 cultivation at 30 °C and 250 rpm. Black arrows denote the mitomycin C- induced putative antibacterial proteins of ~ 30 kDa and ~ 48 kDa. PM and MMC stands for protein marker and mitomycin-C respectively

SDS-PAGE analysis of *Bl* 1951 spontaneously induced putative ABPs exhibited minor bands with the 3–6 h concentrated lysates as compared to *Bl* 1821L (Fig. 5a). Spontaneously induced putative ABPs showed the appearance of ~ 30 kDa and ~ 48 kDa protein bands (black arrows) between 12 and 144 h of *Bl* 1951 growth, after which a decrease of these proteins was observed (Fig. 5a, 5b).

## Discussion

This study reports the spontaneous induction of HMW putative ABPs (bacteriocins) from the insect pathogenic isolates *Bl* 1821L and *Bl* 1951.While assessing the production kinetics of the putative ABPs, it was noted that these ABPs were persistently induced during the course of *Bl* 1821L and *Bl* 1951 growth and affected the growth of producer isolates in liquid after 18 h which was evident from a decrease in the number of viable cells and the results of bioactivity assays. The inherent antibacterial and autocidal bioactivity of the spontaneously induced putative ABPs of *Bl* 1821L and *Bl* 1951 was correlated with the pHs of crude supernatants obtained at various time intervals. Furthermore, SDS-PAGE profile of the spontaneously induced putative ABPs of *Bl* 1821L and *Bl* 1951 at various time intervals was compared with the mitomycin C- induced putative ABPs for the strength of protein banding. Biochemical features including the effect of proteolytic enzymes, pH, and temperature on the bioactivity of crude lysate preparations of the isolates *Bl* 1821L and *Bl* 1951 harbouring the putative phage head (capsid) and tail-like structures were also investigated through disc diffusion assays.

Antibacterial structures, phages and phage-derived bacteriocins, reside in their host bacteria as prophages or defective prophages [10, 70]. These can be induced from the bacterial genomes under the influence of internal (stalled replication forks, reactive oxygen species (ROS)) or external triggers (UV radiation, mitomycin C, ROS, pH, heat) [71, 72] or can be spontaneously induced in the absence of an external trigger [73, 74]. Supernatants of *Bacillus megaterium* lysogens have been found to harbour spontaneously induced phages in the cultivating media under non-inducing conditions [4]. SPI happens due to spontaneous accumulation of DNA damage initiating the host’s SOS response during cell replication and is often accompanied by lysis of the bacterial cells [33, 75]. This important but uncommon phenomenon is taken a detrimental process on the bacterial populations as a small percentage of cells is lost continuously [76, 77]. [78] for the first time reported the simultaneous induction (spontaneous) of F-type and R-type tailocins from the Gram-negative bacterium, *Pragia fontium* 64613. The authors noted the maximum occurrence of spontaneous induction of tailocins after 12 h at 30 °C with heavy shaking and this could be further increased by exposure to UV light at 245 nm or the use of mitomycin C at 1 µg/mL. In the present study, the growth curves of *Bl* 1821L and *Bl* 1951 in all the triplicate experiments did not show strong uniformity and consequently the CFU/mL varied due to unknown factors. However, it was found that the spontaneously induced putative ABPs (bacteriocins) of *Bl* 1951 caused a drastic decline in the number of viable cells (log_10_ CFU/mL) after 18 h of growth which corresponded to the highest antagonistic activity of CFS of this particular time period against the indicator isolates *Bl* 1821L and *Bl* 1951. The pH of *Bl* 1951 CFS extracted after 18 h, like log_10_ CFU/mL and the diameter (mm) of clearing zones (lysis), were statistically significant. Although *Bl* 1821L also experienced a dip in log_10_ CFU/mL after 18 h of growth and more pertinently this period also coincided with the highest antibacterial activity of CFS containing spontaneously induced putative ABPs (bacteriocins), the differences were not significant.

Due to potent antibacterial activity of the CFS of *Bl* 1821L and *Bl* 1951 extracted at various time intervals in bioactivity assays, all the filtered supernatants were subjected to high-speed centrifugation and then ran on SDS-PAGE to visualise the difference between the mitomycin C- induced and the spontaneously induced inhibitory compounds. Previously, we isolated, purified, and characterised the 31.4 kDa and ~ 48 kDa proteins from the mitomycin C-induced cultures of *Bl* 1821L and *Bl* 1951 isolates and determined their antagonistic and autocidal role [26, 61]. In this study, appearance of ~ 30 kDa and ~ 48 kDa protein bands at various time intervals from *Bl* 1821L and *Bl* 1951 also suggested the spontaneous induction of HMW putative ABPs. Furthermore, the bioactivity assays of the spontaneously induced HMW putative ABPs of *Bl* 1951 sustained their autocidal activity across all the evaluated time intervals except in the initial 3–6 h, but for *Bl* 1821L, this was evident even from the early hours of its growth. Typically, bacteria secrete these extracellular antibacterial molecules (bacteriocins) to inhibit growth, kill a wide range of microbial species or just the kin strains, and the producer strains are immune to their lethal effects [16, 79]. However, some members of a genetically identical population of bacteriocins such as *hyicin* 3682 can kill their siblings [80, 81]. [63] whilst determining the antimicrobial potential of the supernatants derived from the isolates *Bl* BGSP7, *Bl* BGSP9, and *Bl* BGSP11 noted the autocidal activity. In addition, the phenomenon is also prevalent among the bacteria that experience a nutritional stress during their growth [82, 83].

Host range (antibacterial spectrum) is the breadth of bacteria (species or strains) that a phage or phage derived bacteriocin can kill [16, 84]. For this purpose, phages or tailocins (R-type) are provided with receptor binding proteins (RBPs) to bind with the surface receptors of a particular host cells to cause lysis [85, 86]. Therefore, it is likely that a slight change in RBPs can alter the antimicrobial spectrum of these antibacterials [87]. [88] demonstrated that switching of a 200 kDa RBP of *Clostridium difficile*, swapped the bactericidal specificity of its R-type tailocins. In this study, PEG 8000 precipitated lysate of *Bl* 1951 harbouring the putative phage tail-sheath protein (~ 48 kDa) exhibited a narrow spectrum of activity that included the kin strains (*Bl* 1821L, *Bl* Rsp, *Bl* CCEB 342, *Bl* NRS 590, *Bl* NCIMB). Similar to this, another entomopathogenic Gram-positive bacterium, *Bacillus pumilus* 15.1, encoding a phage tail-like protein demonstrated its bacteriocin-like activity against the related strains [89]. However, in the past we found a broad spectrum of activity of PEG 8000 precipitated supernatant of *Bl* 1821L including an unrelated strain *C. maltaromaticum* [61]. Typically, phages and PTLBs exhibit a narrow host range [15] but PTLBs with a wide host range have also been reported from the handful strains [90, 91]. Therefore, it is possible that the variation in the host range of PEG 8000 precipitated lysate of *Bl* 1951 and *Bl* 1821L might be due to the differences in the contractile tail sheath RBPs [92]. This variation is critically important for the insect pathogenic strains due to their competition with the other bacteria of the same ecological niche [93, 94].

The expression of genes responsible for the production of AMPs is largely dependent on the temperature and initial pH of the medium [95]. Various metabolic mechanisms like aggregation, adsorption of bacteriocin by producing cells, and proteolytic degradation by specific or non-specific proteases, as well as post-translational modifications to produce active bacteriocins are sensitive to acidification of the cultivating media [96, 97]. [98] reported that pH values decrease gradually over several hours after inoculation due to rapid growth rate of bacteria. The gradual increase in pH of *Bl* 1951 and *Bl* 1821L up to 48 and 60 h of growth aligns with the exponential growth and afterwards a slight fall (stationary phase) where pH values remained almost constant. Typically, at this stage, *Bacillaceae* species produce several organic acids such as malic acid, pyruvic acid, acetic acid, citric acid, succinic acid, α-ketoglutaric acid, propionic acid, and butyric acid [98]. Pyruvic acid is considered as one of the key intermediates in the Tricarboxylic acid (TCA) cycle and Embden-Meyerhof-Parnas (EMP) pathways and plays a vital role in bacteriocin biosynthesis. However, depending on the species and medium composition, the concentration of these organic acids may vary. The time when bacterial growth almost approaches the stationary phase, the concentration of organic acids decreases, as often indicated by a slight increase in fermentation pH [99].

Proteolytic enzymes hydrolyse peptide bonds in substrate proteins, resulting in a widespread, irreversible post-translational modification of the protein’s structure and biological function [100, 101]. Proteinase K is commonly used in molecular biology to digest proteins. Earlier studies have elucidated that bacteriocins from different species can either be activated, inactivated, or do not result in any changes of antimicrobial activity [102, 103]. HMW bacteriocins, BceTMilo and maltocin S16, upon exposure to proteolytic enzymes trypsin, α-chymotrypsin, proteinase K, protease, lipase, and papain, or lysozyme did not lose their killing activity, but α-chymotrypsin caused a 75% loss in bioactivity of maltocin S16 [91, 104]. Proteinase K treatment completely abrogated bactericidal activity of the PTLBs, maltocin P28, and serracin P [105, 106]. LMW bacteriocins of the genus *Brevibacillus* lost their antagonistic activity after treatment with proteinase K [107–109]. In our work, proteolytic enzymes (proteinase K and protease) addition to the crude preparations of *Bl* 1821L and *Bl* 1951 harbouring the HMW putative ABPs abrogated their inhibitory activity, which authenticated their proteinaceous nature.

LMW bacteriocins are mostly heat resistant, but their optimal temperature for the highest antagonism varies depending on species [110, 111]. For example, *Bl* produced bacteriocin such as laterosporulin [109], Bac-GM100 [108], and laterosporulin 10 [107] maintained their thermal stability even after heating at 121 °C for 15–20 min. The vast majority of HMW bacteriocins such as serracin P [105], aquaticin [78], fonticin [78], maltocin P28 [106], and maltocin S16 [104] become inactive upon incubation at a temperature between 45 and 60 °C for 10 min. The crude preparation of the isolates *Bl* BGSP7, *Bl* BGSP9, and *Bl* BGSP11 sustained their stability at 60 °C, 80 °C, 100 °C, and 121 °C heat-treatments for 20 min, although at 121 °C, a decrease in the bioactivity was noted [63]. In our study, the crude lysate of *Bl* 1821 L harbouring the HMW putative ABPs retained stability between 70 and 90 °C and the HMW putative ABPs of *Bl* 1951 tolerated heating up to 100 °C. However, the antagonistic activities of the HMW putative ABPs of *Bl* 1821L and *Bl* 1951 were lost after autoclaving (121 °C) for 15 min.

The genus *Bacillus* bacteriocins are known to be active over a broad range of pH [112]. The crude lysate of the isolates *Bl* BGSP7, *Bl* BGSP9, and *Bl* BGSP11 sustained their stability over a wide pH range (2.0–14.0) [63]. HMW bacteriocin, BceTMilo, killing activity was stable between pH 4.8–8.8, with a 100-fold decrease in activity after 18 h at pH 10.5 and all detectable activity was lost after 18 h at pH 2.0 [91]. However, in our work the crude preparations harbouring the HMW putative ABPs of *Bl* 1821L (pH 2.0–10.0) and *Bl* 1951 (pH 4.0–12.0) sustained their antibacterial activity over a wide pH range. The reduction in cell viability may be speculated from the accumulation of H^+^ ions as reflected from the low pH values which might have perturbed the membrane permeability and caused leakage of some cellular components and the dissipation of the proton motive forces [98, 99]. However, these speculations are subject to experimental validations.

Over the past several years, there has been an expectation to expand the potential of insect pathogenic bacteria beyond their exploration for insecticidal value by making the use of extracellularly released ABPs (bacteriocins) in other avenues like apiculture, crops disease management, food safety, human and animal health etc [113]. Hence, in this context the HMW bacteriocins of insect pathogenic isolates *Bl* 1821L and *Bl* 1951 hold a promising potential as an antibacterial compound. A bibliographical search shows that it is the first report of the spontaneous induction of HMW bacteriocins of *Bl* 1821L and *Bl* 1951 that affected the growth of both the host bacteria after 18 h but the decrease in number of viable cells was significant for *Bl* 1951. This autocidal potency of both the isolates is critically important during production as a potential biopesticide. Using the soft agar overlay method with PEG precipitation, a narrow spectrum of activity was found for *Bl* 1951 precipitated lysate as compared to the previously defined broad spectrum for *Bl* 1821L. This varied antibacterial spectrum of the insect pathogenic *Bl* 1821L and *Bl* 1951 isolates is striking from the interbacterial point of view as it can have serious implications for the survival of host bacterium in natural environments due to the kin exclusion strategy. Furthermore, the crude preparations from the mitomycin C- induced cultures of *Bl* 1821L and *Bl* 1951 harbouring the phage capsid-like and contractile sheath-like structures were biochemically characterised and found to be stable over a wide range of temperatures and pH. This stability of HMW putative ABPs over a wide range of temperatures and pHs can provide an insight into their nature. Overall, our findings added a wealth of knowledge that will be useful in the development of microbial pesticide from the insect pathogenic *Bl* 1821L and *Bl* 1951 isolates.

### Electronic supplementary material

Below is the link to the electronic supplementary material.

Supplementary Material 1

## Data Availability

All data generated or analyzed during this study are included in this published article and its supplementary information file. The insect pathogenic isolates *Bl* 1951 and *Bl* 1821L are deposited in the National Measurement Institute, Melbourne, Australia with the accession NMI No. V12/0001945 (*Bl* 1951) and NMI No. V12/0001946 (*Bl* 1821L). Sequences of *Bl* 1951 (RHPK01000003.1, contig 1) and *Bl* 1821L (NZ_CP033464.1) are deposited under the Bioproject accession number PRJNA503267 in the database of National Center for Biotechnology Information (NCBI), Bethesda, MA, USA.
