# Supplementary material for: Biochemical characterisation and production kinetics of high molecular-weight (HMW) putative antibacterial proteins of insect pathogenic Brevibacillus laterosporus isolates
Source: BMC Microbiol. 2024 Jul 13;24:259. doi: 10.1186/s12866-024-03340-2 (PMC11245835; doi:10.1186/s12866-024-03340-2)
Supplement: Supplementary file 1 — Supplementary Material 1 [file 12866_2024_3340_MOESM1_ESM.pdf]

**Supplementary Information (SI)**

**BMC Microbiology**

# Biochemical characterisation and production kinetics of high molecular-weight (HMW) putative antibacterial proteins of insect pathogenic *Brevibacillus laterosporus* isolates

**Tauseef K. Babar*[1,2], Travis R. Glare[1,3], John G. Hampton[1,3], Mark R. H. Hurst[4], Josefina Narciso[1,3]**

[1] Bioprotection Research Centre, Lincoln University, Lincoln 7647, Canterbury, New Zealand

[2] Department of Entomology, Faculty of Agricultural Sciences and Technology, Bahauddin Zakariya University, Multan 60000, Pakistan

[3] Faculty of Agriculture and Life Sciences, Lincoln University, Lincoln 7647, Canterbury, New Zealand

[4] Resilient agriculture, AgResearch, Lincoln Research Centre, Christchurch, New Zealand

* **Correspondence:** tauseefkhan@bzu.edu.pk

**Author for correspondence:** Dr. Tauseef Khan Babar, Assistant Professor, Department of Entomology, Faculty of Agricultural Sciences and Technology, Bahauddin Zakariya University, Multan 60000, Pakistan

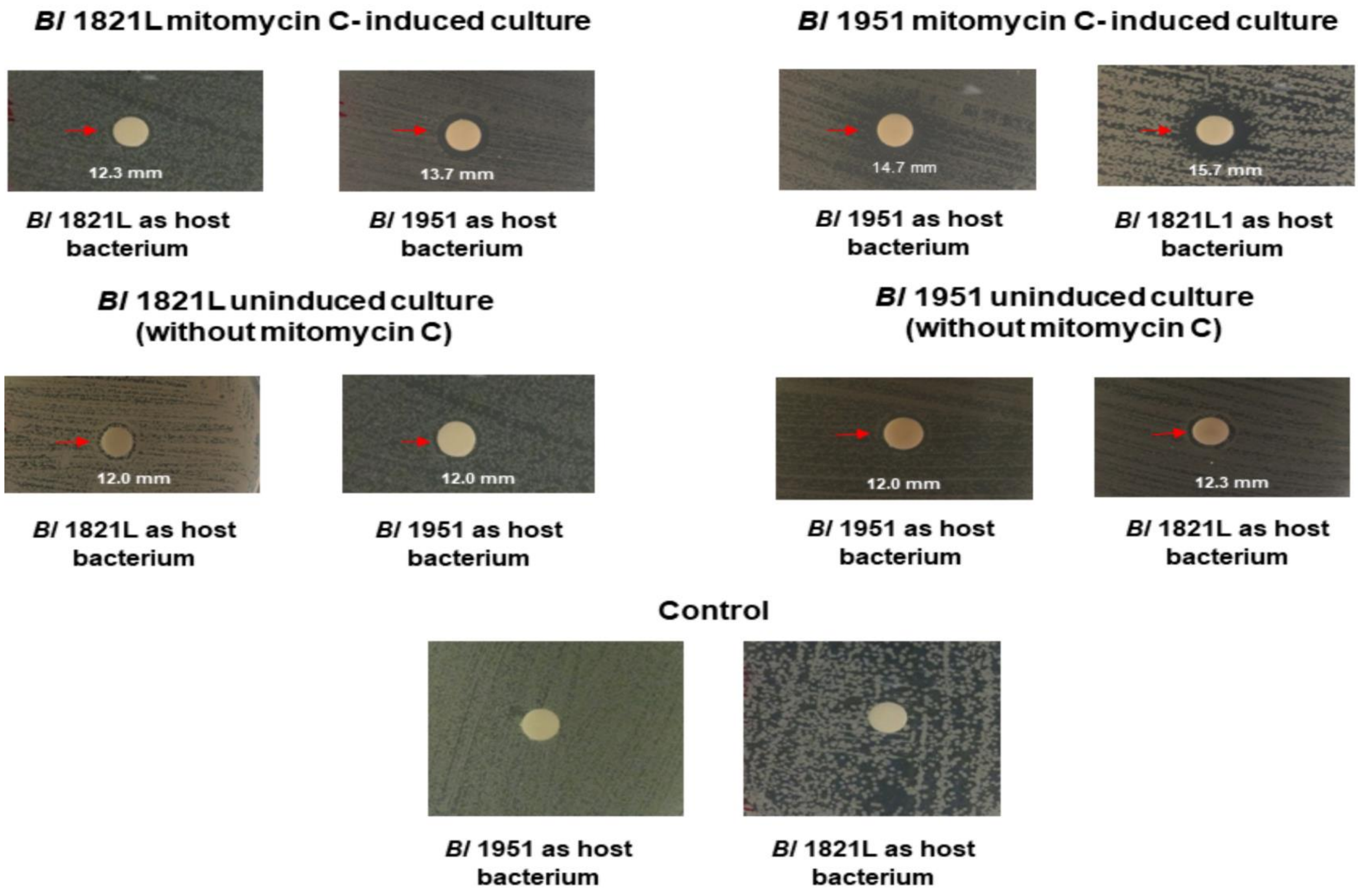


**Fig. S1** Disc diffusion assay test of mitomycin C- induced and uninduced (without mitomycin C) cultures of *Bl* 1821L and *Bl* 1951 against the producer and vice versa isolate. The red arrows denote the zone of inhibition produced on the lawns of the indicator isolates due to bioactivity of the putative antibacterial proteins (ABPs)

Note: Cultures of *Bl* 1821L and *Bl* 1951 isolates without addition of mitomycin C served as a control. Cell free supernatant (CFS) of the control treatment (without mitomycin C) was obtained and assayed for bioactivity similar to the mitomycin C- induced cultures

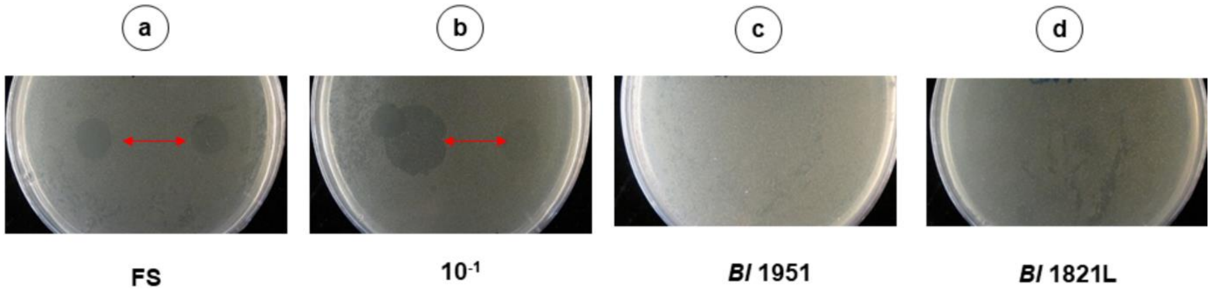


**Fig. S2** Antagonistic activity of *Bl* 1951 filtered supernatant after polyethylene glycol (PEG) 8000 precipitation in serial dilution assay against the indicator isolate *Bl* 1821L. The red arrows denote the lysis zones developed on the lawns of *Bl* 1821L isolate due to the antagonistic activity of PEG 8000 precipitated antibacterial proteins of *Bl* 1951

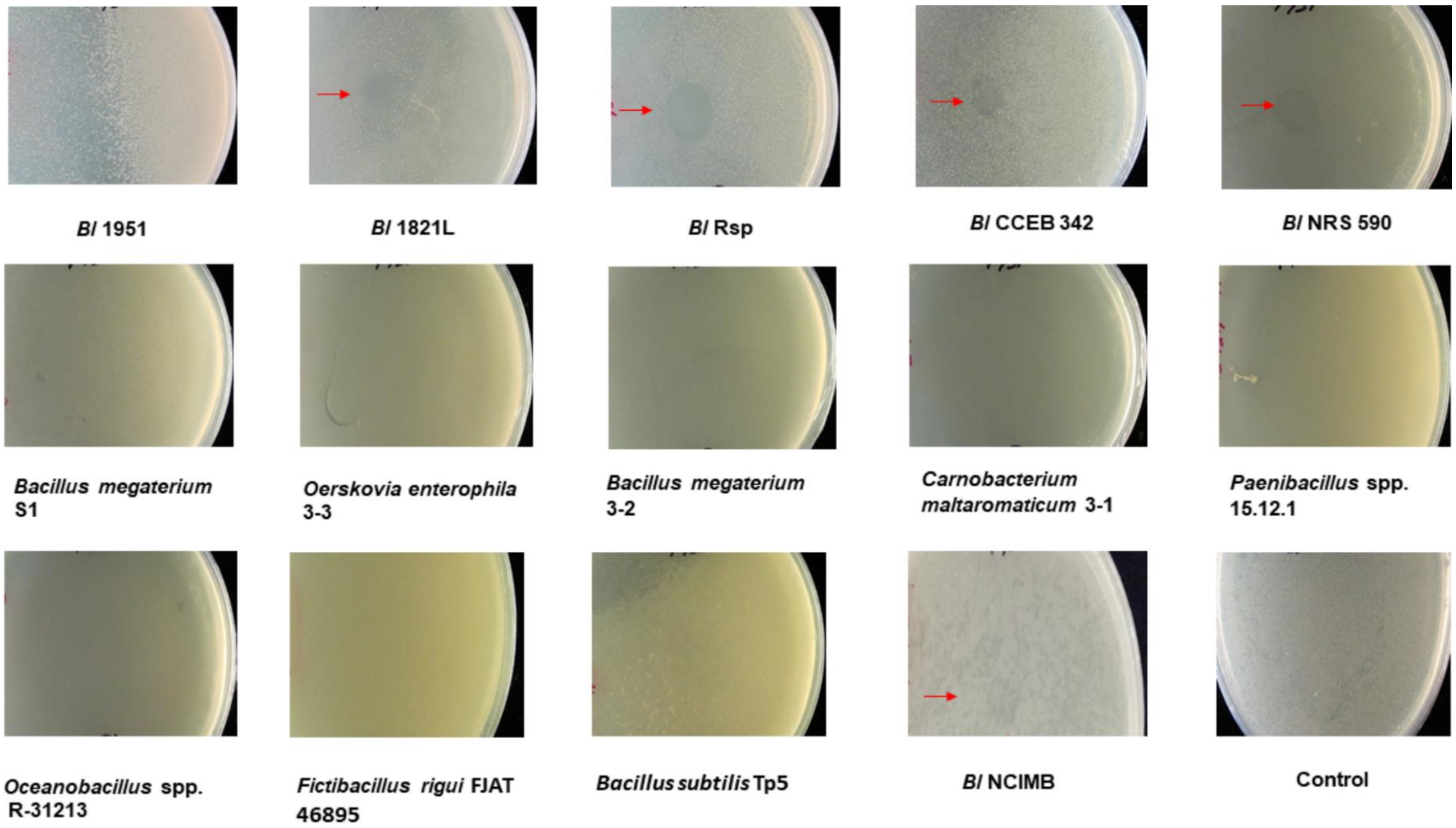


**Fig. S3** Antibacterial activity of mitomycin C- induced culture CFS of *Bl* 1951 after PEG 8000 precipitation against various Gram-positive bacteria. The red arrows denote the zone of inhibition produced on the lawns of host bacteria due to the putative antibacterial activity of PEG 8000 precipitated filtrate of *Bl* 1951

**Table S1. Effect of enzymes on the bioactivity of filtered supernatant of *Bl* 1821L and *Bl* 1951 harbouring the putative antibacterial proteins (bacteriocins) against the indicator isolates *Bl* 1951 and *Bl* 1821L**

| **Enzymes** | **Zone of inhibition** | |
| --- | --- | --- |
| | ***Bl* 1951 as the host bacterium** | ***Bl* 1821L as the host bacterium** |
| Proteinase- K | - | - |
| Protease | - | - |
| Catalase | + | + |
| Control (Mitomycin C) | + | + |

- = No zone of inhibition
+ = Zone of inhibition

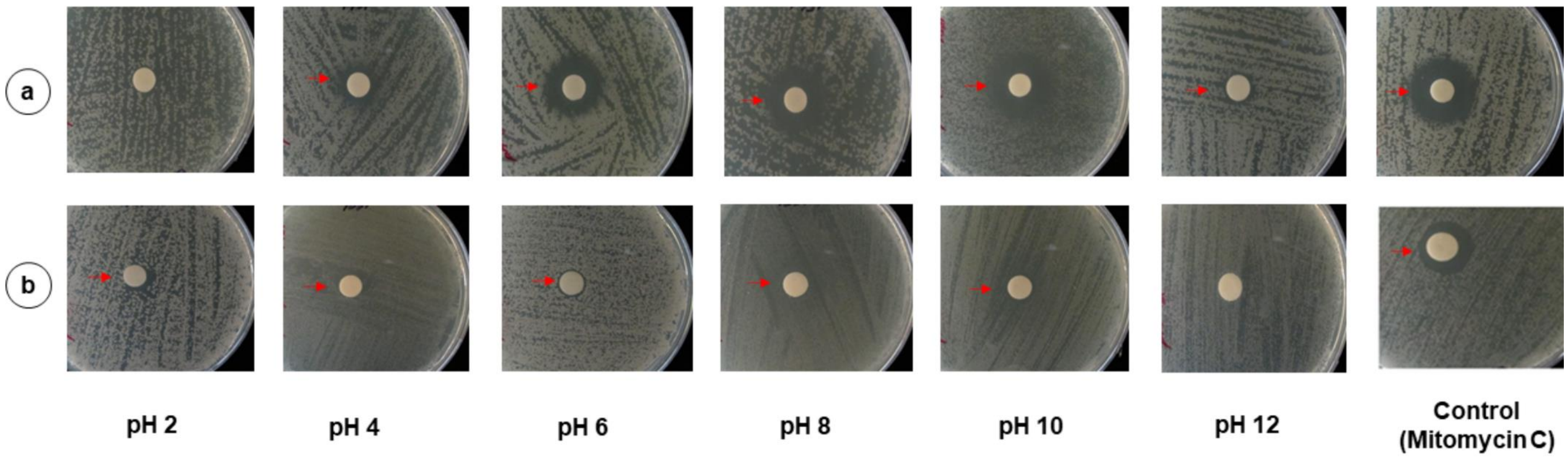


**Fig. S4** Bioactivity assay of the effect of pH on the bioactivity of crude putative ABPs of *Bl* 1951 (a) and *Bl* 1821L (b) against the indicator isolates *Bl* 1821L and *Bl* 1951. The red arrow denote the zones of inhibition produced on the lawns of indicator isolates due to the bioactivity of putative ABPs

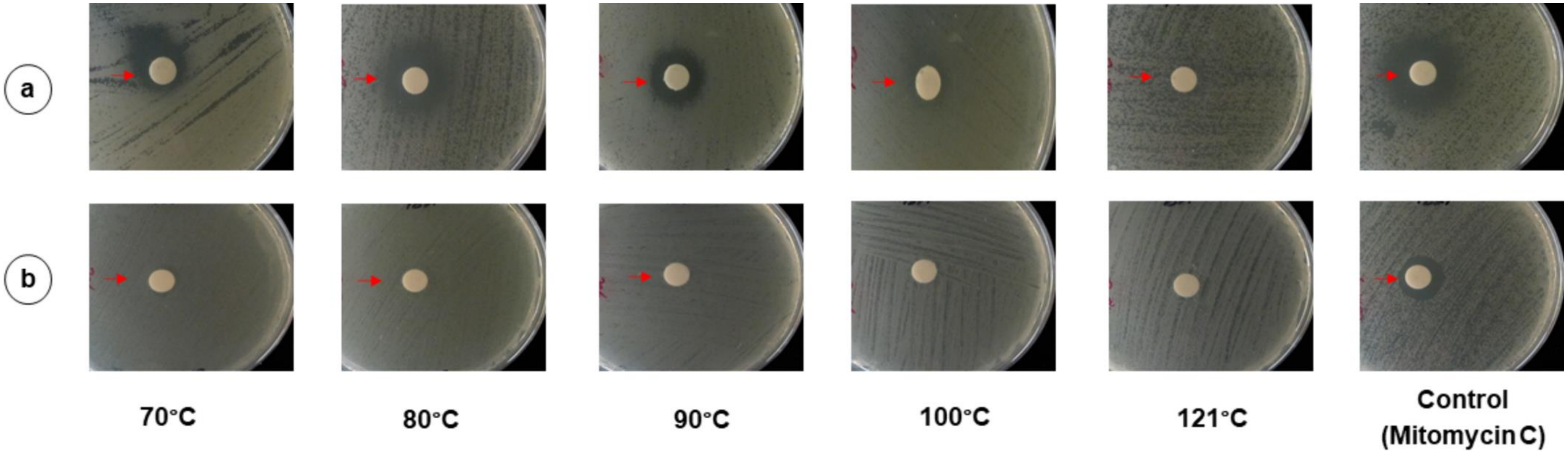


**Fig. S5** Bioactivity assay of the effect of different temperatutres on the bioactivity of crude putative ABPs of *Bl* 1951 (a) and *Bl* 1821L (b) against the indicator isolates *Bl* 1821L and *Bl* 1951. The red arrow denote the zones of inhibition produced on the lawns of indicator isolates due to the bioactivity of putative ABPs

**Table S2. Production kinetics of *Bl* 1821L spontaneously induced putative antibacterial proteins (bacteriocins) at various time intervals and assay test of crude cell free supernatant (CFS) against the indicator isolates *Bl* 1821L and *Bl* 1951**

| Time interval (Hours) | $\log_{10}$ CFU/mL | pH of CFS | Zone of inhibition diameter (mm) | |
|---|---|---|---|---|
| | | | ***Bl* 1821L as the host bacterium** | ***Bl* 1951 as the host bacterium** |
| 3 | 5.576 | 7.06 | 11.33 | 10.78 |
| 6 | 5.518 | 7.04 | 11.67 | 11.67 |
| 12 | 5.304 | 8.01 | 13.33 | 14.33 |
| 18 | 4.960 | 8.35 | 13.22 | 15.22 |
| 24 | 5.280 | 8.69 | 13.11 | 15.22 |
| 36 | 5.384 | 9.12 | 13.67 | 15.33 |
| 48 | 5.796 | 9.26 | 12.33 | 13.78 |
| 60 | 5.995 | 9.37 | 13.00 | 15.67 |
| 72 | 5.859 | 9.31 | 12.78 | 15.44 |
| 96 | 5.626 | 9.42 | 11.67 | 13.89 |
| 120 | 5.929 | 9.47 | 12.11 | 13.45 |
| 144 | 6.166 | 9.33 | 12.56 | 12.78 |
| 168 | 6.138 | 9.32 | 13.11 | 12.56 |
| 192 | 6.440 | 9.32 | 12.56 | 12.22 |
| 216 | 6.243 | 9.40 | 12.89 | 11.44 |
| 240 | 6.241 | 9.39 | 11.67 | 12.22 |
| *LSD (5%) | 0.636 | 0.317 | 2.221 | 1.922 |

*LSD= Least significant difference

Note: Results were considered significant if the difference in the mean value of the assessed parameters was more than the value of least significant difference (LSD) at 5%

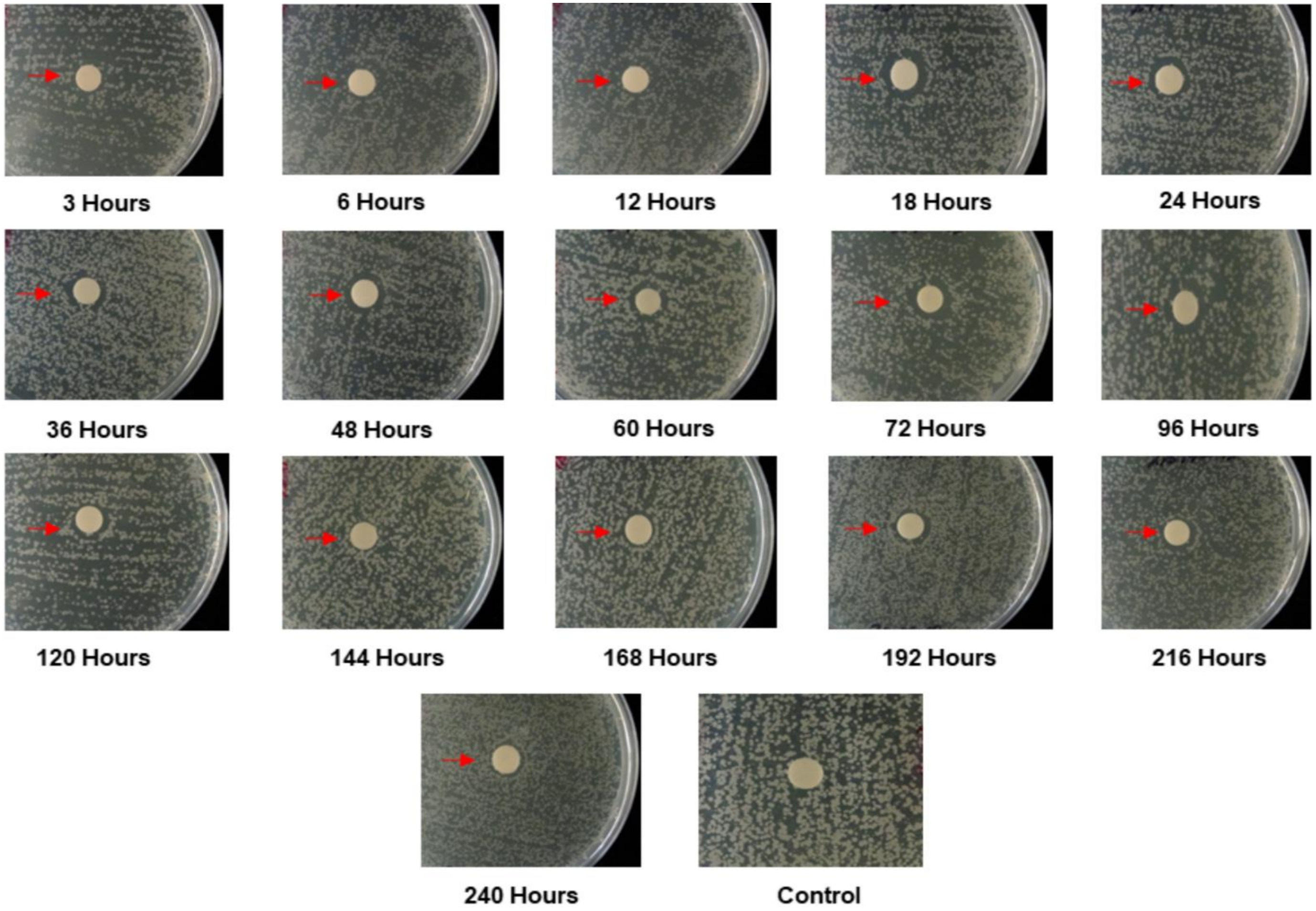


**Fig. S6** Bioactivity assay of spontaneously induced putative ABPs crude supernatant of *Bl* 1821L extracted at various time intervals of its growth at 30°C and 250 rpm against the indicator isolate *Bl* 1821L. The red arrows denote the zone of inhibition produced on the lawns of *Bl* 1821L due to the bioactivity of putative ABPs of *Bl* 1821L

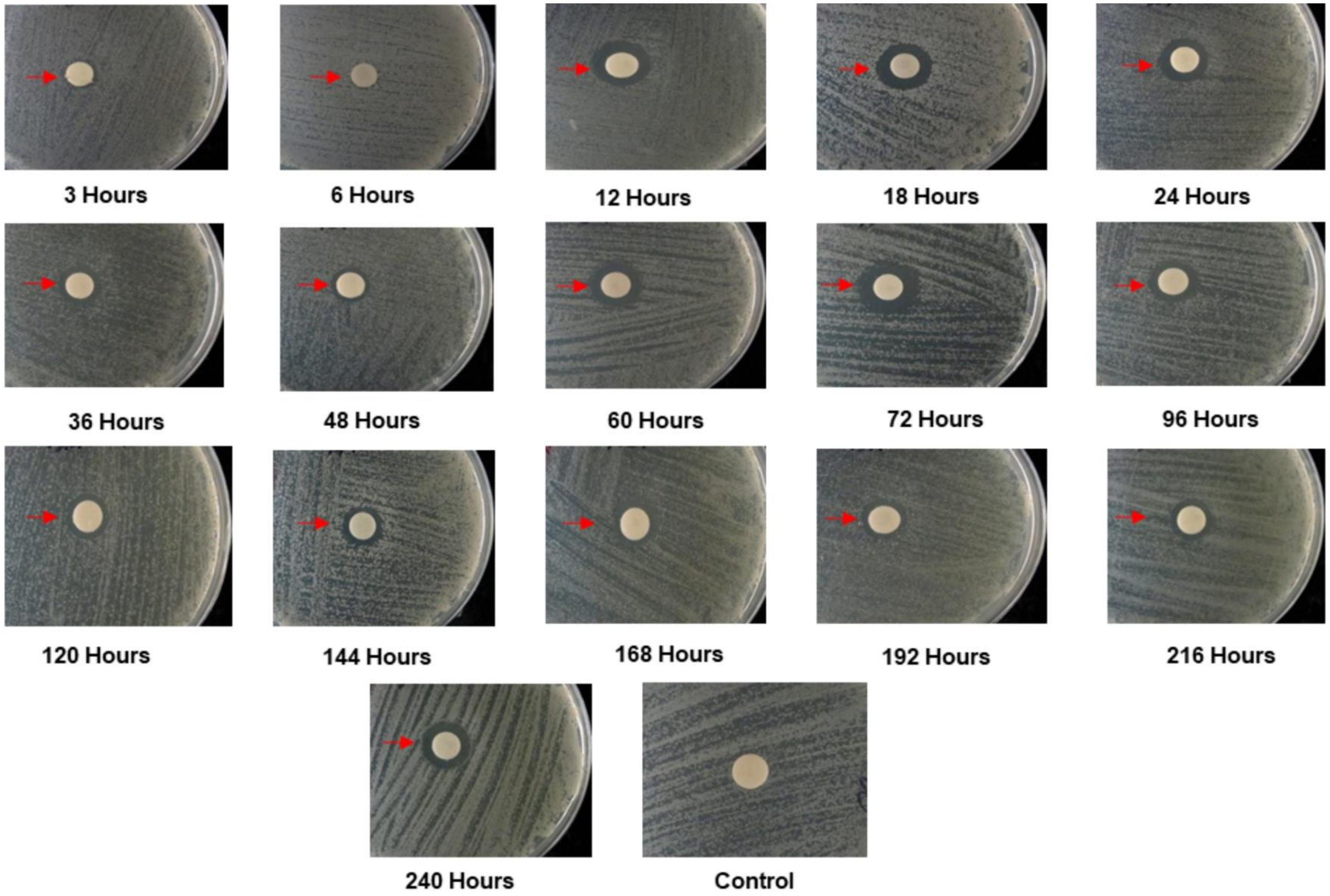


**Fig. S7** Bioactivity assay of spontaneously induced putative ABPs crude supernatant of *Bl* 1821L extracted at various time intervals of its growth at 30°C and 250 rpm against the indicator isolate *Bl* 195l. The red arrows denote the zone of inhibition produced on the lawns of *Bl* 1951due to the bioactivity of putative ABPs of *Bl* 1821L

**Table S3. Production kinetics of *Bl* 1951 spontaneously induced putative antibacterial proteins (bacteriocins) at various time intervals and assay test of crude cell free supernatant (CFS) against the indicator isolates *Bl* 1951 and *Bl* 1821L**

| Time interval (Hours) | $log_{10}$ CFU/mL | pH of CFS | Zone of inhibition diameter (mm) | |
|---|---|---|---|---|
| | | | *Bl* 1951 as the host bacterium | *Bl* 1821L as the host bacterium |
| 3 | 6.239 | 6.95 | 0.00 | 12.33 |
| 6 | 6.315 | 7.08 | 0.00 | 12.22 |
| 12 | 6.868 | 7.68 | 10.67 | 13.56 |
| 18 | 6.156 | 8.18 | 13.33 | 15.00 |
| 24 | 6.000 | 8.61 | 11.89 | 13.11 |
| 36 | 6.574 | 9.05 | 11.67 | 13.11 |
| 48 | 7.079 | 9.08 | 12.67 | 12.33 |
| 60 | 6.795 | 9.12 | 12.22 | 11.56 |
| 72 | 6.812 | 9.18 | 12.44 | 12.67 |
| 96 | 6.968 | 9.24 | 13.78 | 13.11 |
| 120 | 7.151 | 9.23 | 12.78 | 12.89 |
| 144 | 7.103 | 9.24 | 12.22 | 12.78 |
| 168 | 7.044 | 9.28 | 12.00 | 14.67 |
| 192 | 6.988 | 9.29 | 13.11 | 13.67 |
| 216 | 7.086 | 9.32 | 11.89 | 13.67 |
| 240 | 7.253 | 9.20 | 12.78 | 13.56 |
| *LSD (5%) | 0.444 | 0.165 | 1.268 | 2.067 |

*LSD= Least significant difference

Note: Results were considered significant if the difference in the mean value of the assessed parameters was more than the value of least significant difference (LSD) at 5%

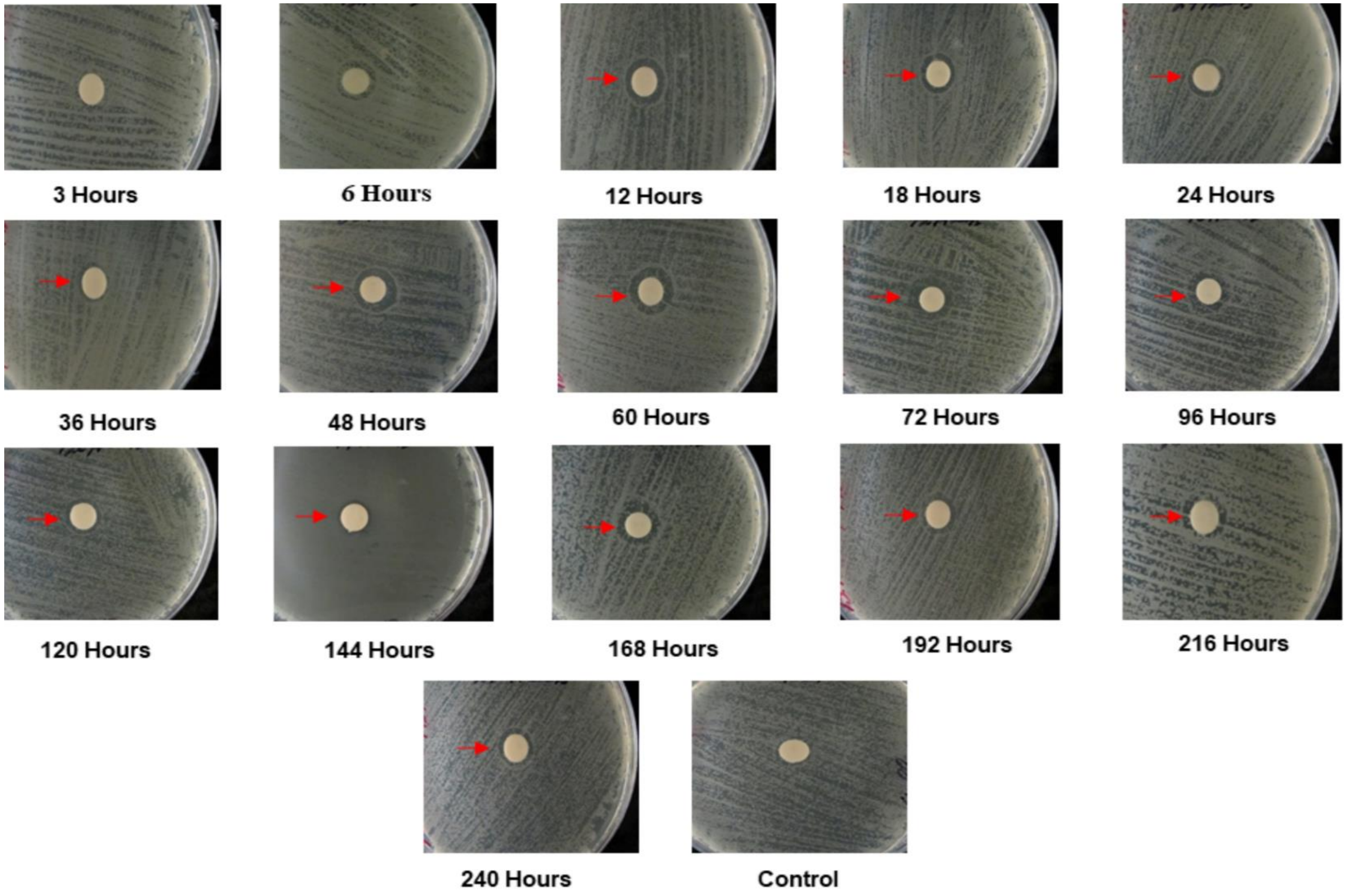


**Fig. S8** Bioactivity assay of spontaneously induced putative ABPs crude supernatant of *Bl* 1951 extracted at various time intervals of its growth at 30°C and 250 rpm against the indicator isolate *Bl* 195l. The red arrows denote the zone of inhibition produced on the lawns of *Bl* 1951 due to the bioactivity of putative ABPs of *Bl* 1951

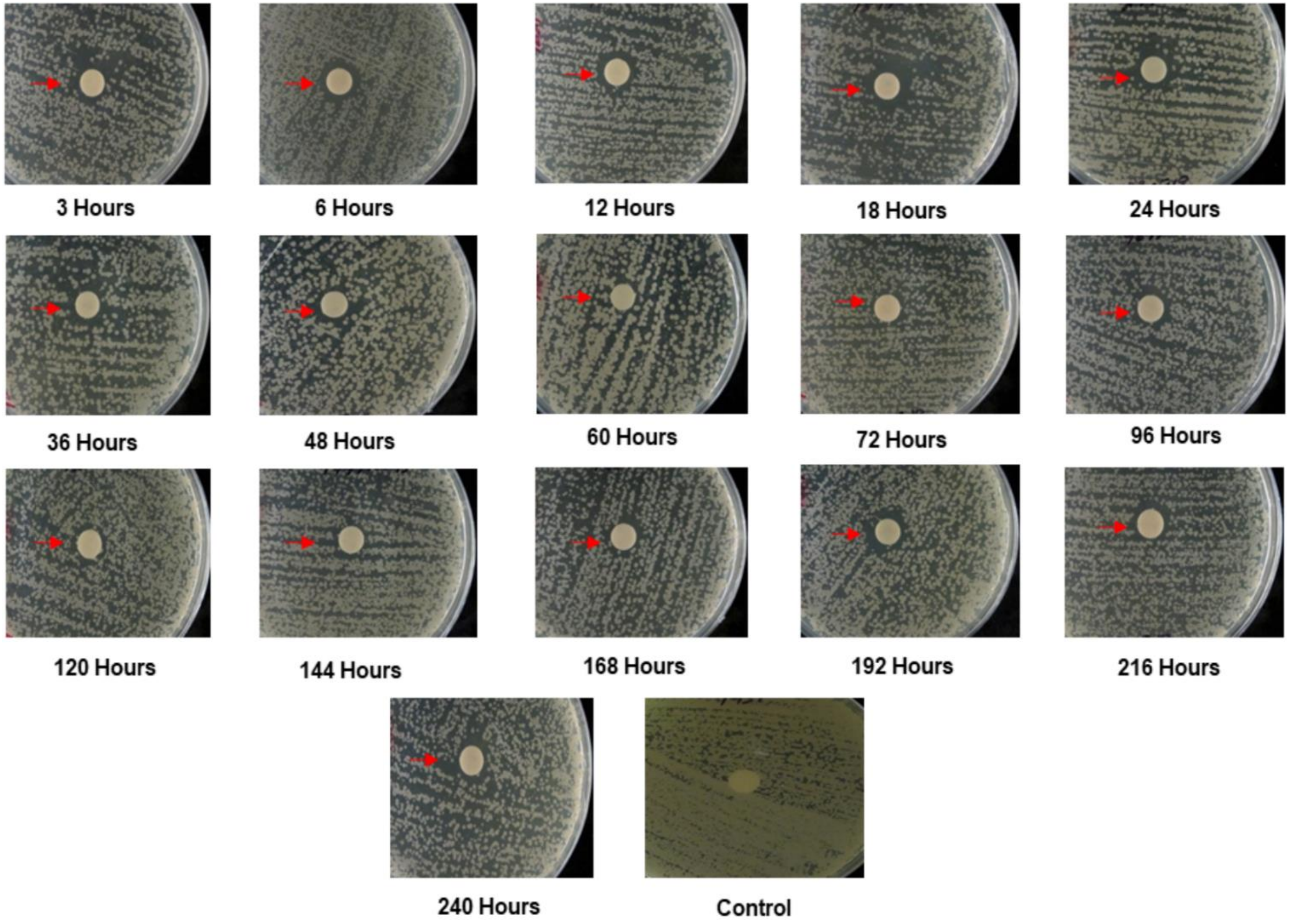


**Fig. S9** Bioactivity assay of spontaneously induced putative ABPs crude supernatant of *Bl* 1951 extracted at various time intervals of its growth at 30°C and 250 rpm against the indicator isolate *Bl* 1821L. The red arrows denote the zone of inhibition produced on the lawns of *Bl* 1821L due to the bioactivity of putative ABPs of *Bl* 1951
